# Social instability is associated with an elevated stress response but not with a fitness cost across vertebrate studies

**DOI:** 10.1098/rsos.250691

**Published:** 2025-07-23

**Authors:** Adriana A. Maldonado-Chaparro, Liam R. Dougherty, Loren D. Hayes, Luis A. Ebensperger

**Affiliations:** ^1^ Escuela de Ciencias e Ingeniería, Universidad del Rosario, Bogota, Colombia; ^2^ Max Planck Institute of Animal Behavior, Konstanz, Germany; ^3^ Department of Evolution, Ecology and Behaviour, Institute of Infection, Veterinary and Ecological Sciences, University of Liverpool, Liverpool, UK; ^4^ Department of Biology, Geology, and Environmental Science, University of Tennessee at Chattanooga, Chattanooga, TN, USA; ^5^ Facultad de Ciencias Biológicas, Pontificia Universidad Católica de Chile, Santiago, Chile

**Keywords:** group instability, group turnover, meta-analysis, social isolation, social organization, social structure

## Abstract

Studies on single species often support that social instability influences physiological stress responses and individual fitness within social groups, yet the underlying mechanisms and adaptive consequences remain unclear. We conducted a meta-analysis spanning from 1970 to 2025, incorporating data from 59 articles across avian and mammalian species, to investigate the effects of social instability on stress and fitness. We found a positive association between social instability and glucocorticoid levels, consistent with our expectation of physiological response. Fitness declined with increasing social instability, but this relationship was not statistically significant and did not support our expectations. We found no statistically significant moderating effects of social system component, sex, age, taxonomic group and study type (experimental versus observational) on either stress or fitness outcomes. However, females and adults exhibited stronger positive stress correlations and stronger negative fitness correlations, and observational studies showed a similar trend when compared with experimental studies. Our results highlight a significant gap in the literature and call for greater taxonomic diversity and increasing use of experimental field studies to better understand the effects of social instability. Our meta-analysis further stresses the need for improved study standardization, as less than 20% of the publications examined were suitable for analysis.

## 1. Background

Social instability, characterized by sudden or persistent changes in group composition or the dynamics of social relationships, is a natural phenomenon in group-living animals. These changes often result from demographic events that lead to increased uncertainty within the group and can negatively affect individual physiology [1] and fitness [2]. Social instability can impact different components of animal social systems [3]. Instability in social organization (size and composition of groups) may result when individuals die [4–7], disperse from a group of origin to join and/or breed in a different group [8–13], or are evicted by socially dominant individuals [14–16]. Instability in social organization may also be a consequence of group fission [17–19]. Instability in social structure (patterns of interactions among group members [3]) may arise from changes in within-group social associations (e.g. alliances [20]) or from changes in within-group interactions (e.g. dominance relationships [21]). Recently, there has been an increase in the number of empirical studies evaluating the effects of social instability on behaviour, physiology and fitness across a wide diversity of species. However, quantitative analyses to better understand the underlying mechanisms and adaptive consequences of social instability are lacking.

A major focus of research has been to determine the mechanisms underlying the effects of instability in social organization and social structure, including activation of the hypothalamic-pituitary-adrenal [HPA] axis—a stress-response mechanism maintaining homeostasis [22,23]. Short-term elevations of glucocorticoids (such as cortisol or corticosterone) above baseline levels are associated with predictable demands contingent to life history stages [24,25]. Elevated glucocorticoids are beneficial during stressful conditions because they are involved in physiological effects that shift energy from non-essential processes to processes critical for immediate survival. Likewise, elevated glucocorticoids may be needed to sustain lactation [26] or to produce an additional litter [27]. Under these conditions the stress response is adaptive. However, repeated or extended exposure to elevated glucocorticoids can be costly and may result in metabolic demands that exceed available resources [28,29]. Sustained glucocorticoid levels can also lead to ‘wear and tear’ resulting in immunosuppression [29,30] and disruption of negative feedback mechanisms that deactivate the HPA response [29,31]. These costs would reduce an individual’s ability to respond to environmentally or socially mediated stressors and lead to increased disease risk and lower reproductive success [24,32–34]. In natural populations, the negative consequences of elevated glucocorticoids on individuals may vary based on numerous factors, including sex, condition, duration of exposure to stressors and social status [26,35–38].

To date, studies on HPA responses to social instability have shown varied results. Elevated glucocorticoid levels are associated with individuals transferred to different groups in female feral horses (*Equus caballus*) and female meerkats (*Suricata suricatta*) [39,40] but not so with immigration of male howler monkeys (*Alouatta pigra*) [41]. Similarly, glucocorticoid levels increase significantly in resident male woolly monkeys (*Lagothrix lagotricha*) [42] and resident female baboons (*Papio hamadryas ursinus*) [43] after facing immigration of one or more males but not so in male sifakas (*Propithecus verreauxi*) [44]. Consistency and disparity in effects are similarly evident among biomedical and animal welfare-oriented studies. For instance, groups of hens (*Gallus gallus*) experiencing the replacement of the most socially dominant member are more aggressive, have elevated glucocorticoid levels and decrease egg production [45]. In contrast, female goats (*Capra hircus*) in socially unstable groups increase aggression but do not alter levels of glucocorticoids or offspring growth [46]. These examples suggest that the effects of social instability may be contingent on factors such as taxonomic affiliation, sex or age of subjects examined, or the type of study conducted (e.g. lab- versus field-based).

Studies have also aimed to determine the impact of social instability in social organization and structure on the fitness of individuals. For example, dispersal alters group composition and social dynamics, contributing to instability and affecting fitness components across different species. Reproductive success decreases in dispersing females of feral horses [47,48], western gorillas (*Gorilla gorilla*) [49] and female brown jays (*Cyanocorax morio*) [12], yet increases in male and female Arabian babblers (*Turdoides squamiceps*) [50] or does not change in male and female long-tailed tits (*Aegithalos caudatus*) [51]. Such variation is also evident among group residents. While turnover of group members decreases reproductive success of breeding female greater anis (*Crotophaga major*) [52] and Alpine marmots (*Marmota marmota*) [2], the replacement of male or female breeders does not affect infant survival of owl monkeys (*Aotus azarai*) [53]. Examinations of the effects of instability in social structure are limited, yet those available suggest greater consistency. For instance, mare feral horses that maintain relatively more stable and long-term relationships with other non-kin females enhance reproductive success through reducing aggression received [54]. Similarly, male bottlenose dolphins (*Tursiops aduncus*) that maintain stronger and evenly distributed social bonds with other males over time (i.e. stable) attain higher reproductive success [55]. In degus (*Octodon degus*), the offspring that experience a stable group composition grow faster than offspring experiencing the daily removal of their mother [56]. This evidence suggests that the effect of social instability may also be contingent on the component of the social system examined. Yet, a more systematic examination of available evidence is needed to determine if the functional consequences of unstable social organization are different from those linked to unstable social structure.

To broadly understand the consequences of social instability, we used meta-analytic tools to quantify how socially unstable conditions (i.e. social instability) impact the physiological response to stress (e.g. activation of the HPA axis) and direct fitness measures (e.g. reproductive success of impacted individuals, and growth and survival of impacted individuals and their offspring). We asked: (i) do socially unstable conditions induce elevated HPA-stress response? (ii) are there fitness costs associated with socially unstable conditions? (iii) do they differ depending on whether social organization or social structure is affected? (iv) do they differ with sex (iva) or age (ivb) of individuals experiencing socially unstable conditions? (v) do they differ across taxa? and [vi] do they differ between experimental and observational studies?

We hypothesize that social instability activates the HPA response, thus stimulating stress-related hormone production that can influence an individual’s physiological status and potentially its fitness. We predicted [1] overall, HPA hormone levels (ACTH, glucocorticoids) are positively associated with socially unstable conditions and that this trend is more positive with increasing duration of social instability. If chronically elevated glucocorticoid levels have negative consequences, then we assume that social instability is associated with fitness costs (but see [24]). Thus, we also predicted that [2] direct fitness measures decrease with increasing social instability. Although social relationships (i.e. social structure) are strongly correlated with fitness across different species [57] the effects of instability are likely contingent on the extent to which groups include one or more key individuals or are characterized by group members assuming different roles. Therefore [3], we cannot predict the directionality of the effect whenever instability examined affected social structure compared with social organization. Given that sex- and age-related differences in the response to stress are highly variable [58,59], we did not expect the HPA responses to social instability to vary between (iv.a) sexes and (v.a) ages. However, the reproductive success of females is more sensitive to social changes than males [60], especially in non-biparental species [48,61] because of their reliance on social bonds [62]. Therefore, we expected (iv.b) fitness measures to show a stronger negative correlation in females than males. Also, juveniles may have more time or resilience to adjust to new social conditions and still breed successfully later as adults. As such, we expected (v.b) a stronger correlation in adults than in juveniles. Given the overall focus on avian and mammalian species by researchers addressing these questions, we also included phylogenetic relationships. However [5], we did not predict *a priori* phylogenetic differences. Finally, because these questions have been addressed with different research approaches, we also considered the type of experimental approach used and expected [6] a larger correlation in experimental than in observational studies. Based on our findings, we recommend key priorities for future studies.

## 2. Methods

We performed a systematic review following the reporting guidelines of the PRISMA (i.e. Preferred Reporting Items for Systematic Reviews and Meta-Analyses) method [63].

### 2.1. Search strategy

To identify research articles on social instability, we conducted a literature search of peer-reviewed journal articles published between January 1970 and December 2024 using four databases: Web of Science Core Collection, Pubmed, Scopus and Google Scholar. We standardized the search strategy for each database (see next paragraph). Additionally, we considered articles from a previous search conducted by the authors in 2018 and identified other eligible studies, which initially did not appear during the database searches. Although we added our relevant articles to the analysis at the end of the process, we did not consider publications previously found as duplicates. We limited our search to peer-reviewed original research articles in English, Spanish or French. Our literature search was conducted in three steps, first in 2019 (which did not include Google Scholar results) and then updated on January 12th, 2023 (including Google Scholar results) and on January 21st, 2025 (which did not include Google Scholar results). At this stage, we identified a set of related concepts and keywords to include the range of variations these concepts exhibit in the literature in our search terminology. This approach allowed us to effectively construct queries that include this diversity, ensuring a thorough search strategy. For a full description of the strategy, see electronoic supplementary material, Appendix S1.

For the literature search, we used multiple combinations of key terms depending on the dataset, and different search queries for each of them. For Web of Science, we included: (TS=(‘rank $stability’ OR ‘group $stability’ OR ‘social $stability’) AND WC=(‘Ecology’ OR ‘Zoology’ OR ‘Biodiversity Conservation’ OR ‘Behavioral Sciences’ OR ‘Evolutionary Biology’ OR ‘Biology’ OR ‘Ornithology’ OR ‘Biodiversity Conservation’)), for PubMed we included: ‘social instability’ OR ‘social stability’ OR ‘social instability’ OR ‘social stability’ OR ‘group instability’ OR ‘group stability’, for Scopus we used: ‘social instability’ OR ‘social stability’ OR ‘social instability’ OR ‘social stability’ OR ‘group instability’ OR ‘group stability’ and for Google Scholar we considered: ‘social instability’ OR ‘social stability’ OR ‘rank stability’ OR ‘rank stability’ OR ‘group instability’ OR ‘group stability’ AND animal. Although we focused on two aspects of the social system (social organization and social structure), we kept the search broad to maximize the identification of pertinent studies.

### 2.2. Article screening

First, we exported the results of each search from PubMed, Scopus and Web of Science to an EndNote readable file. Results from Google Scholar searches were retrieved and saved for further analyses using the online software ‘publish or perish’ [64]. Second, we created a single EndNote library with all the records obtained from the four searches. In EndNote, we automatically identified and removed the duplicates and exported the resulting titles, abstracts, author and year of publication to a csv file. Finally, we sorted the file by author’s last name, year, title and manually identified any remaining duplicates.

The initial screening based on titles and abstracts was performed by trained students who worked under the supervision of A.A.M.-C. and L.D.H. Irrelevant articles were excluded and then we retrieved full texts of the remaining articles using EndNote. A.A.M.-C. and L.D.H evaluated the selected studies based on the inclusion and exclusion criteria independently and discussed any differences of opinion on the inclusion of articles. A PRISMA diagram summarizing the literature search and study screening process is included in electronic supplementary material, figure S1.

To be considered for inclusion, a study had to satisfy the following criteria:

(1) Be a peer-reviewed scientific article presenting original empirical data.
(2) Consider a group-living (non-human) animal species.
(3) Report some aspect of fitness or stress response following a change in social instability, in terms of either social organization (size and composition of social groups) or social structure (patterns of social interactions among conspecifics).
(4) Present sufficient statistical information to allow for calculation of an effect size (see next section).

We excluded research articles that were not empirical (e.g. review articles, theoretical modelling). We included empirical studies on mammals, birds and fish in the wild, semi-natural and laboratory, regardless of sex or age. Studies conducted over multiple time points were considered only if statistics and sample sizes of the most recent time point measured were reported. In these cases, we analysed the baseline point (before instability) and the first point following the instability event, excluding later points where the system may have returned to a stable state. We included studies comparing groups that differed in their degree of social instability, as well as studies testing the same set of individuals before and after a change in social instability. We considered multiple indices of the physiological stress response, including levels of ACTH (a tropic hormone regulating the secretion of glucocorticoids) and primary glucocorticoid hormones (e.g. cortisol, corticosterone). Since studies focused on integrated responses, we also recorded ‘general glucocorticoid levels’ when reported. Additionally, we considered potential downstream effects of elevated HPA activity, including adrenal weight and damage to the gastric lining. Similarly, we considered multiple indices of fitness, including body weight (or change in body weight over time) of adults, reproductive success, body size of offspring and the number of injuries sustained.

### 2.3. Data extraction and effect size calculation

We extracted the following data from all suitable research publications: focal species taxonomic order, genus and species, field of study (biomedical, functional), type of study (laboratory, semi-natural, field), social system component subject to instability (social organization, social structure), duration of instability, age category (adult, adolescent = refer to non-dependent offspring, or both), sex (female, male, or both) response category (fitness, physiological) and the author who extracted these measures in each published study examined. We also extracted all available statistical information, including total sample size, sample size per treatment and control, statistics and standard errors (s.e.) or standard deviations (which we transformed to s.e.). When the statistical data were not provided in the main text or supplementary material, but we could recover it from figures, we used the software WebPlotDigitizer [65] to extract the raw data from figures. Given that not all studies reported consistently individual-level data, we conducted all analyses at the group-level. A detailed, full listing of the data extracted is provided in the full database (electronic supplementary material). Note that some of this list is more detailed than the list of moderators ([5] in total), as we used this information to characterize each of the studies fully screened in our analysis.

Once the data were extracted, we calculated effect sizes. We used the correlation coefficient *r* as the effect size for the analysis [66]. In this analysis, the effect size quantifies the strength of the relationship between social instability and a given response measure. The correlation ranged from 1 to −1, and a negative effect size means that instability reduces the fitness of impacted individuals (smaller offspring body weight, slower offspring weight gain, fewer offspring, longer offspring development, smaller offspring, more injuries), or leads to a reduced HPA-stress response (lower glucocorticoid levels). Based on this coding and on predictions 1 and 2, we expected a significant negative correlation between social instability and fitness, and a significant positive correlation between social instability and the HPA-stress response. Effect sizes were either taken directly from the text, converted from other reported test statistics (using equations presented in [66]), or calculated directly using raw data extracted from figures.

### 2.4. Statistical analysis

All analyses were performed using R v4.1.2 [67] and the Metafor package v3.4 [68]. We performed separate analyses for HPA-stress responses and fitness measures. Analyses for each dataset followed the same methods.

First, we assembled a phylogenetic tree for the species represented in each dataset to account for the possibility that closely related species exhibit similar effect sizes. To do so, we used the R packages rotl v3.0.12 [69] and ape v5.5−2 [70] to build a supertree using the available phylogenetic and taxonomic information in the Open Tree of Life database [71]. Branch lengths for this tree were first set to 1 and then made ultrametric using Grafen’s method [72]. The tree and its standardized branch lengths were then converted into a variance–covariance matrix for incorporation into the meta-analysis models, using the vcv function in ape. We then created two pruned trees, including only the species used in the HPA-stress response or fitness data subsets, using the drop.tip function in ape.

Second, we ran intercept-only multilevel meta-analysis models to determine the average correlation for each dataset [73]. Multilevel models were used to account for several potential sources of non-independence between correlations: non-independence due to multiple correlations coming from the same study, non-independence due to multiple correlations from the same species and non-independence due to shared phylogenetic history [74]. We determined the importance of each by sequentially adding them as random factors to the intercept-only model and assessing model fit using the Akaike Information Criterion (AIC; electronic supplementary material, table S1). The best-fitting model for the HPA-stress-response dataset included study ID as the only random factor. The best-fitting model for the fitness dataset included both species ID and study ID as random factors. Phylogeny was incorporated into all models using a variance–covariance matrix. All models also included observer ID as a random factor and were needed to properly calculate residual variance [75]. We used the Fisher’s *z* transformation of the correlation coefficient (*zr*) as the response variable in all models, as this results in a distribution that is approximately normal [66].

We considered a mean correlation to be significantly different from zero whenever the estimated 95% confidence interval did not include the zero. We quantified heterogeneity for the overall model (total *I^2^*), and for each random factor in the model by means of using the *I^2^* statistic [73,76]. *I*^2^ values of 25, 50 and 75% were considered low, medium and high, respectively [76].

Third, we tested whether the average correlation of each dataset was influenced by seven categorical moderators:

(1) The social system component (social organization SO, social structure SS, or both): we do not have an *a priori* expectation of differences in the effect of instability on social structure compared with social organization.
(2) The physiological response traits: (i) indicators of elevated stress (or potential wear and tear)—fell into one of four trait categories: ACTH, corticosterone, cortisol and general glucocorticoids. Only two studies measured adrenal weight [77,78], and one measured damage to the gastric lining [79], for a total of five effect sizes. We therefore excluded these downstream effects from the final analysis. (ii) Fitness responses fell into one of four trait categories: body weight, reproductive success, injury or offspring fitness.
(3) The sex of focal individuals (male, female, both sexes combined or not specified): we expect a stronger effect of instability for females compared with males for fitness but not for HPA hormones (ACTH and glucocorticoids).
(4) The age (adult, adolescent or both) of focal subjects: we expect a stronger effect for adults and juveniles with respect to fitness but not for HPA hormones.
(5) The taxonomic group (primates, rodents, other mammals or birds; one fish correlation was excluded from this comparison). We do not have an *a priori* expectation of differences due to phylogenetic relationships.
(6) Experimental design (experimental, observational). We expect stronger correlation in experimental than in observational studies.
(7) Duration of instability. We expect a stronger effect for socially unstable conditions that lasts longer.

To do this, we ran a series of meta-regressions. Each model included the same random factors as the best-fitting intercept-only model indicated previously but also included a single moderator as a categorical fixed factor. For all meta-regressions, we first removed any categories with fewer than five data points. For the instability duration analysis, we also removed four outliers that were longer than 200 days (three effect sizes for stress response, one for fitness), as these points are likely to have high leverage in a regression analysis. We determined whether the moderator influenced the average correlation using the *Q_M_* statistic. For each model, we quantified the amount of variation explained by the fixed factor (marginal *R^2^*) using the orchaRd package [80].

Fourth, we tested for two sources of publication bias. We first tested whether the average correlation changes over time [81] by running a meta-regression with publication year as a continuous fixed factor. Additionally, we tested whether studies with smaller sample sizes are less likely to be published [66], using a meta-regression with study precision as a continuous fixed factor [82]. Meta-regressions included the same random effects as for the best-fitting intercept-only models above. For model summaries, see electronic supplementary material, tables S2 and S3.

## 3. Results

The search strategy returned a total of 8249 records (Web of Science = 263; PubMed = 4983; Scopus = 2018; Google Scholar = 985) plus 69 records from a previous search. After removing duplicate records (*n* = 7794) and the first screening conducted we had a total of 364 papers. From this, 306 did not meet our criteria when evaluating the full-text read-through and 13 had to be further excluded because they were not accessible [4], were duplicates [5] or were not relevant [4]. These results, including reasons for exclusion, are reported in electronic supplementary material. A total of 59 research articles met our criteria for inclusion, all of which had the accessible data needed for the meta-analysis.

### 3.1. Do individuals exhibit elevated hypothalamic-pituitary-adrenal-related stress responses under socially unstable conditions?

**Figure 1.**
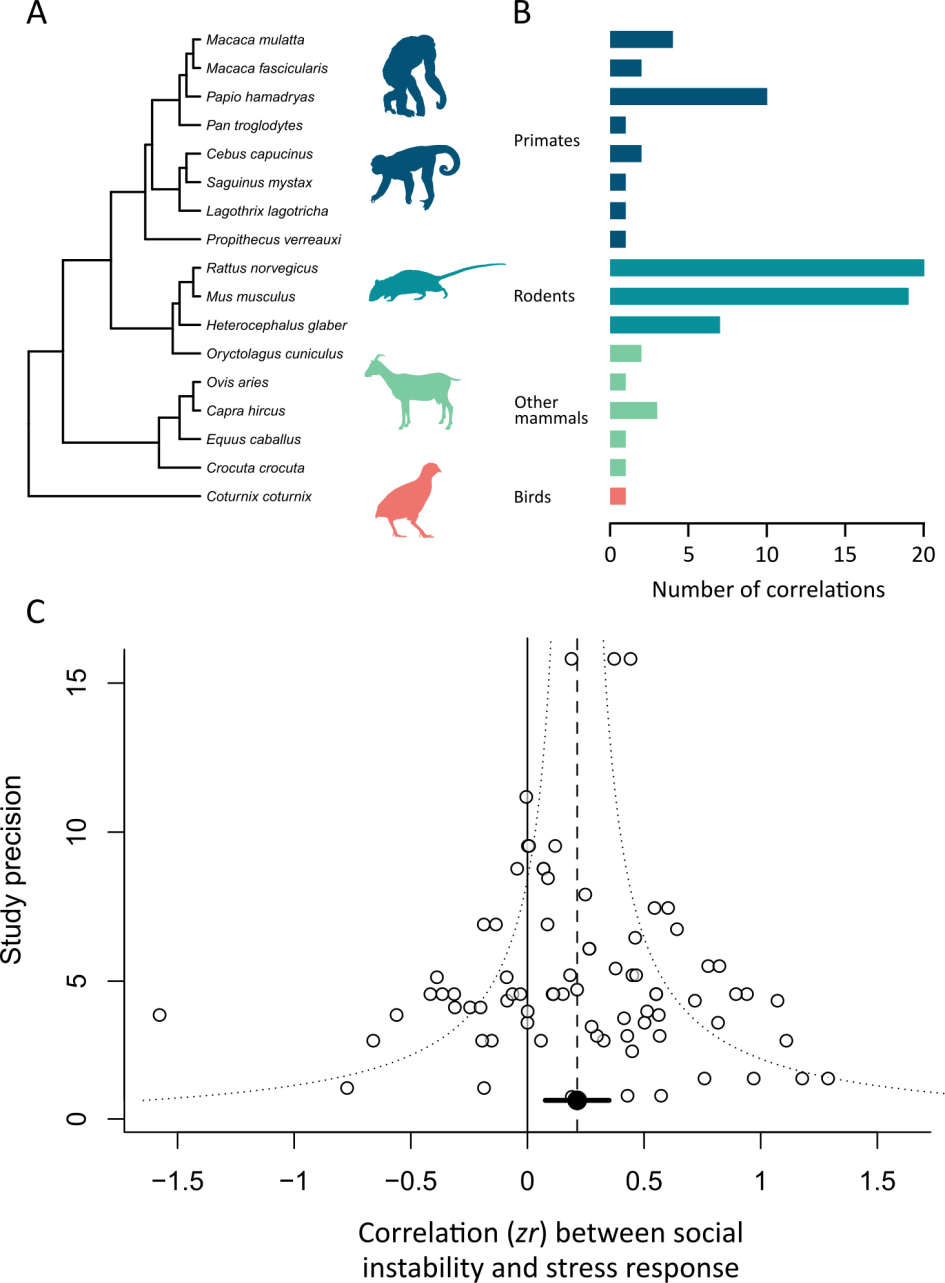
Results for the stress-response meta-analysis. (A) Phylogenetic tree showing the 17 species, with representative species silhouettes. (B) Histogram showing the number of correlations for each species. (C) Funnel plot showing the relationship between effect size (*zr*: Fisher’s z transformation of the correlation coefficient) and the inverse standard error (a measure of study precision- larger values represent studies with larger sample sizes, *n* = 77 correlations). The dashed line shows the overall mean effect size estimate from the meta-analysis model. The dotted line illustrates the typical expected ‘funnel’ shape, with effect sizes from studies with large sample sizes resulting in estimates that are closer to the mean.

We obtained 77 stress-response correlations from 40 studies. Of 17 species (figure 1A), eight were from primate species, three from rodents, five from other mammals and one was from birds. Rodents contributed the most data points, making up 59% of the dataset (*n* = 46; figure 1B). Almost a third of correlations (*n* = 24) came from studies on Norway rats (*Rattus norvegicus*) (figure 1B). Studies measured four types of HPA hormone response: corticosterone (*n* = 33), cortisol (*n* = 17) and its regulatory hormone ACTH (*n* = 7) and general glucocorticoid levels (*n* = 20).

The best-fitting model for the stress-response dataset included study ID and observer ID as random effects (electronic supplementary material, table S1). Species ID and phylogenetic history explained a negligible amount of variation and so were removed from the model. The dataset was characterized by high heterogeneity (total *I^2^* = 84.6%), with most being explained by study ID (*I^2^* = 81.2%). Overall, social instability led to significantly increased stress responses (mean *r* = 0.21, 95% CI = 0.076–0.336, *k* = 77; figure 1C).

### 3.2. Are there fitness costs associated with socially unstable conditions?

**Figure 2.**
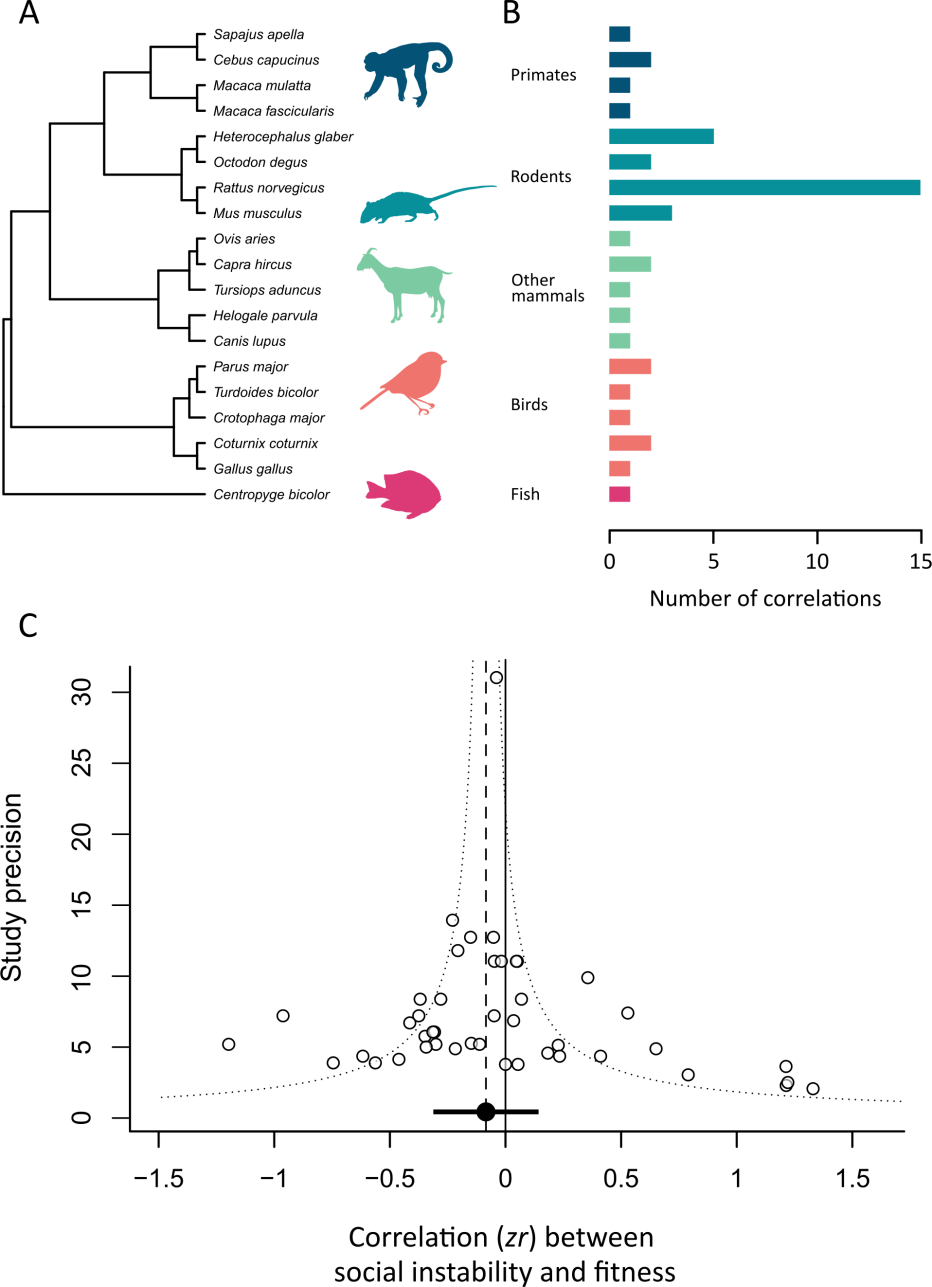
Results for the fitness meta-analysis. (A) Phylogenetic tree showing the 19 species, with representative species silhouettes. (B) Histogram showing the number of correlations in the dataset of each species. (C) Funnel plot showing the relationship between effect size (*zr*: Fisher’s *z* transformation of the correlation coefficient) and the inverse standard error (a measure of study precision—larger values represent studies with larger sample sizes, *n* = 44 correlations). The dashed line shows the overall mean effect size estimate from the meta-analysis model. The dotted line illustrates the typical expected ‘funnel’ shape, with effect sizes from studies with large sample sizes resulting in estimates that are closer to the mean.

We obtained 44 fitness correlations from 29 studies. Of 19 species (figure 2A), four were from primate species, four from rodents, five from other mammals, five from birds and one from a fish. Rodents contributed the most data points, making up more than half of the dataset (*n* = 25; figure 2B). Over a third of correlations (*n* = 15) came from the Norway rat (figure 2B). Fitness was measured in terms of body mass or weight gain (*n* = 27), adult reproductive success (*n* = 14), offspring fitness (*n* = 2) and the number of injuries (*n* = 1).

The best-fitting model for the fitness dataset included species ID, study ID and observer ID as random effects (electronic supplementary material, table S1). Phylogenetic history explained a negligible amount of variation and so was removed from the model. The dataset was characterized by high heterogeneity (total *I^2^* = 93.7%), with most being explained by species ID (*I^2^* = 80.6%) and the remaining explained by study ID (13.2%). There was not a statistically significant relationship between social instability and fitness for the full dataset (mean *r* = −0.084, 95% CI = −0.293 to 0.132, *k* = 44; figure 2C).

### 3.3. Do correlations with stress and fitness differ depending on which aspect of the social system is changed?

Most studies examined the effects of changing social organization (60 of 77 correlations for stress response, 37 of 44 correlations for fitness). The average correlation recorded did not depend on whether social organization or social structure was changed for either the stress response (*Q_M_* = 0.64, *p* = 0.73, *R^2^* = 0.02) or fitness (*Q_M_* = 0.1, *p* = 0.75, *R^2^* = 0.002).

### 3.4. Do correlations with stress and fitness depend on the sex or age of individuals experiencing socially unstable conditions?

Studies were roughly equally likely to examine the effects of social instability in either females or males (stress response: 36 females, 36 males, 5 both; fitness: 22 females, 17 males, 5 both). The average correlation did not depend on the sex of subjects for stress response (*Q_M_* = 1.91, *p* = 0.39, *R^2^* = 0.04) or fitness (*Q_M_* = 4.69, *p* = 0.1, *R^2^* = 0.07). Most studies examined the effects of social instability in adults (65 of 77 correlations for stress response, 35 of 44 correlations for fitness). The average correlation did not depend on the subject age category (adult vs juvenile) for stress response (*Q_M_* = 0.01, *p* = 0.75, *R^2^* = 0.004) or fitness (*Q_M_* = 0.001, *p* = 0.99, *R^2^*<0.001).

### 3.5. Do the correlations with stress and fitness vary across taxa?

The average correlation was not contingent on the taxonomic group for stress response (*Q_M_* = 0.52, *p* = 0.77, *R^2^* = 0.01) or fitness (*Q_M_* = 3.61, *p* = 0.31, *R^2^* = 0.16).

### 3.6. Do the correlations with stress and fitness differ depending on the type of study?

Social instability was experimentally manipulated in most studies examining stress response (58 of 77 correlations) and fitness (33 of 44 correlations). The average correlation did not depend on the type of study for stress response (*Q_M_* = 1.44, *p* = 0.23, *R^2^* = 0.04) or fitness (*Q_M_* = 3.85, *p* = 0.05, *R^2^* = 0.14).

### 3.7. Publication biases

We verified that the earliest included study was from 1990 for both the stress response and direct fitness datasets [83]. We found no evidence that the mean correlation for stress response has changed over time (*Q_M_* = 1.13, *p* = 0.29, *R^2^* = 0.03; electronic supplementary material, figure S2A), or that studies with small sample sizes were less represented in either dataset (stress response: *Q_M_* = 0.14, *p* = 0.7, *R^2^* = 0.002; fitness: *Q_M_* = 0.04, *p* = 0.84, *R^2^* < 0.01). The mean correlation for fitness has decreased over time (*Q_M_* = 4.62, *p* = 0.03, *R^2^* = 0.18; electronic supplementary material, figure S2B).

### 3.8. Response trait

The average correlation did not depend on which response trait was reported, for stress response (*Q_M_* = 0.62, *p* = 0.89, *R^2^* = 0.01; electronic supplementary material, figure S3A) or fitness (*Q_M_* = 0.79, *p* = 0.37, *R^2^* = 0.03; electronic supplementary material, figure S3B).

### 3.9. Instability duration

The average correlation did not depend on the duration of instability, for stress response (*Q_M_* = 0.29, *p* = 0.59, *R^2^* = 0.004; electronic supplementary material, figure S4A) or fitness (*Q_M_* = 1.4, *p* = 0.24, *R^2^* = 0.06; electronic supplementary material, figure S4B).

## 4. Discussion

Our study revealed that social instability weakly and positively affects HPA hormone levels and has no consistent effect on fitness. We did not find evidence highlighting the influence of moderating factors examined, such as sex, age, taxa or social system components, on correlations with social instability. There was no evidence of a publication bias in the analysis of sociality and HPA-stress response. However, there was a bias in the analysis of social instability and fitness, most likely due to the over-representation of one publication [84], and a trend towards reporting reproductive success in recent years. Since instability in one or several components of the social system may result in interactive effects [85], we suggest that future studies should distinguish between the effects of instability in each component on stress physiology and examine other components of the social system.

In support of our hypothesis, results confirmed an overall positive association between social instability and the HPA-stress response across the range of taxa examined. The relationship between social instability and HPA-stress-related hormones is complex and varies depending on various factors such as physiological state and life history stage [86,87]. The magnitude of the stress response should be correlated with the expected cost of the stressor. For example, increased glucocorticoid levels may signal poor condition resulting from prolonged or repeated stressors and/or pathology [24,88]. Socially unstable conditions could also impact negative feedback mechanisms and adrenomedullary responses [89], which in turn, could contribute to pathologies such as cardiovascular damage [90]. The stress response also involves metabolic dysregulation leading to oxidative stress and associated pathologies [91,92]. Alternatively, elevated glucocorticoids could indicate generally positive responses, such as increased HPA responsivity to potential threats to survival and reproduction or a reproductively successful state [27]. In this case, we expect positive associations between both social instability and HPA responses and HPA responses during socially unstable conditions and fitness. The effect may also be dependent on the duration of socially unstable conditions, which was not detected in our analysis. Thus, future meta-analytical studies require that we tease apart the different mechanisms by which social instability impacts the stress response.

Elevated glucocorticoids may also be associated with different components of the social system. For example, there is evidence that some individuals experience elevated glucocorticoids at high population density [93] or during territorial intrusions [94]. Our analysis did not account for the potential effects of rank-related (i.e. social structure) responses to social instability because only a handful of studies provided appropriate data to do so (e.g. [95]). Limited evidence, mostly from primate studies, suggests that an individual’s response to social instability depends on an individual’s dominance status, but in a sex-specific manner [96–99]. For example, dominant male primates typically exhibit elevated levels of glucocorticoid hormones compared with subordinate males during periods of rank instability, whereas the opposite trend occurs during periods of social stability [96]. In contrast, dominant females tend to have lower levels of glucocorticoids than subordinates during periods of rank instability, particularly in cases in which the dominance status is maternally inherited [96,100]. Exceptions to these patterns could be associated with species-specific costs and benefits of acquiring and maintaining social positions in a hierarchy and how dominance status is achieved [35,96,101–104]. Rank (and ecological conditions) can also mediate the fitness consequences of instability. For example, a long-term study of olive baboons (*Papio anubis*) supported that female miscarriage rates are highest among high-ranking pregnant females exposed to males rapidly gaining social status during periods of high rainfall, (ii) but independently of female rank when rainfall is low [105]. Moreover, sex- and rank-related stress responses during periods of social instability may also depend on how recently a new group has been formed in primates [106]. Furthermore, the lack of sufficient individual-level data on rank limits our understanding of the rank-related effects of social instability and the individual-level consequences, highlighting the need for future studies in this area.

The social environment can also reduce stress levels [35], thus altering the levels of HPA-stress-related hormones, through social support or buffering. The presence of bonded partners has been demonstrated to lower glucocorticoid levels in canids [107], primates [108] and piglets [109], indicating that social interactions can mitigate socially stressful conditions. In primates, social relationships (sometimes referred to as ‘friendships’) represent a coping strategy to psycho-physiological stress. These bonds have been associated with increased longevity among adults and improved offspring survival [62,110,111]. Friendships with resident males have been shown to reduce the likelihood of injury and death of offspring during takeovers by immigrant males [112]. Female baboons that form friendships have lower average glucocorticoid levels during periods of immigrant male take-over [113] and smaller mean increases in glucocorticoids during periods of immigrant male attack on their offspring [114] than females without male friends. Lactating females with close male associates (or bonded males) may perceive a lower risk of infanticide to their offspring, thereby reducing the negative consequences of long-term elevation of glucocorticoids as well as the cost of instability. Friendships also enhance grooming, a coping strategy that reduces physiological stress [115]. These studies show how affiliative social relationships can significantly influence an individual’s response to social instability. Thus, it was surprising that our analysis did not detect differences associated with social organization and social structure. It is possible that the lack of a significant effect size was a consequence of limits in data quality, such as the general lack of information on the quality and number of social relationships an individual has within a social unit. This highlights the importance of reporting descriptive data on social organization and social structure [116].

Contrary to our initial expectations, we found no supporting evidence for an effect of social instability on fitness. While we found a negative trend between social instability and fitness measures that agreed with the frequently negative effects reported by specific studies, this relationship was not statistically significant. This result is intriguing because social instability and HPA activation seem to be associated across taxa. This begs the question, ‘Why is there a consistent stress response to social instability that does not strongly or uniformly impact fitness?’ Fitness consequences of social instability may vary with characteristics such as demography (age, sex), individual condition (e.g. stress levels, reproductive state), social system components (social structure, social organization) [60,85] and ecological conditions (e.g. parasite load, food abundance, ambient temperature and population density) [117]. Understanding how social instability shapes fitness may require a more nuanced examination of the potentially confounding factors at play. We need more studies reporting glucocorticoid-fitness relationships within the same populations experiencing periods of stable and unstable social conditions to determine the mechanistic role played by the HPA system in underlying the fitness consequences of social instability.

Of the 7764 studies initially identified, only 364 (less than 5%) were in the context of social instability, and only 59 studies were deemed suitable for our analysis. This highlights the novelty of our meta-analysis and the lack of research on the impact of social instability on individuals. The year bias in the analysis of social instability and fitness might have been a consequence of this small sample size and a heterogeneous dataset. Priorities in research on social instability should be to expand taxonomic diversity and increase the number of experimental field studies that allow for the determination of causation under natural conditions. Data on the adrenomedullary system and androgen hormone production during periods of social instability [118] are required to better understand how social instability influences stress physiology. Finally, a comprehensive understanding of the consequences of social instability will require examining how behavioural and physiological responses influence mating behaviours [119] and maternal care [56].

Our results also highlight the need for standardized protocols for investigating the effects of social instability, thus ensuring comparability or complementarity across biomedical and functionally oriented publications. A determination of whether the HPA responses of individuals reflect a cost or an adaptive response to social instability will require more integrative, empirical studies reporting relationships between instability and HPA activity and instability and fitness from individuals in the same population. These studies also need to standardize reporting duration and intensity of social instability (stressor) and HPA responses. These protocols should include information on the dominance rank, the number and quality of social relationships and the residency status of the focal individuals. Including these variables would allow us to disentangle the effects of other known mediators of the effects of instability, such as early life experience, social support and dispersal [39,98,108,120]. Our study also stresses the importance of improving reporting and reproducibility, two critical aspects leading to stronger comparative studies and an overall higher quality of scientific research [121].

In conclusion, our meta-analysis supported the hypothesis that social instability is stressful but not the hypothesis that social instability leads to fitness costs. Data on other potentially mediating factors, such as quantity and quality of social relationships, dominance rank and ecological conditions, were not analysed because they were not reported in the suitable publications used in this study. The inclusion of these data in future meta-analyses should enhance the detectability of potential context-dependent effects. Our study highlights the need for a more comprehensive understanding of these complex relationships to unravel the effects that social instability can have on individuals across taxa.

## Data Availability

The full dataset of the screened articles, the raw searches and the code for analysis are provided in the Online Supplementary Material [122]. Supplementary material is available online [123].
